# Genes on bovine chromosome 18 associated with bilateral convergent strabismus with exophthalmos in German Brown cattle

**Published:** 2008-09-22

**Authors:** S. Fink, S. Mömke, A. Wöhlke, O. Distl

**Affiliations:** Institute for Animal Breeding and Genetics, University of Veterinary Medicine Hannover, Foundation, Hannover, Germany

## Abstract

**Purpose:**

Bilateral convergent strabismus with exophthalmos (BCSE) is a widespread inherited eye defect in several cattle populations. Its progressive condition often leads to blindness in affected cattle and shortens their length of productive life. Furthermore, breeding with BCSE-affected animals is forbidden by the German animal welfare laws. We performed a mutation and association analysis for three candidate genes (troponin T type 1 [*TNNT1*], retinol dehydrogenase 13 [*RDH13*], and TCF3 fusion partner [*TFPT*]), which are located within the previously identified BCSE-linked region on the telomeric end of bovine chromosome 18 (BTA18). In addition, we developed single nucleotide polymorphisms (SNPs) within these three candidate genes and nine other genes that are contained in this genomic BCSE-region to perform association analyses with BCSE in German Brown cattle.

**Methods:**

We performed cDNA analyses of all three candidate genes using eye tissues of three affected German Brown cows and three unaffected controls. Furthermore, we screened the exonic and the adjacent genomic sequences of *RDH13*, *TNNT1*, and *TFPT* using four BCSE-affected and four controls of German Brown cattle. Here, we included all exons of *RDH13* and those exons of *TNNT1* and *TFPT* for which SNPs were detected by cDNA analyses. In addition, we developed 21 polymerase chain reaction (PCR) products for 17 more genes in the BCSE region and searched them for polymorphisms. All markers detected were genotyped in 48 BCSE-affected German Brown cows and 48 breed and sex matched controls and tested for association with BCSE.

**Results:**

In total, we detected 29 SNPs in 12 genes. In the coding sequence of the three candidate genes, we identified 10 exonic SNPs and a new splice variant of *TNNT1*. Four SNPs were associated with the BCSE phenotype in single marker-trait analyses. These SNPs were located within *DHDH* (dihydrodiol dehydrogenase dimeric), *CPT1C* (carnitine palmitoyltransferase 1C), *TNNT1*, and *NALP7*. The marker-trait association for haplotypes including five SNPs of *CPT1C*, *SYT5* (synaptotagmin V), *RDH13*, and *NALP7* (NLR family, pyrin domain containing 7) revealed a significant association with BCSE. We identified three individual haplotypes that were significantly associated with BCSE. These haplotypes spanned the region from 56.05 Mb to 62.87 Mb on BTA18.

**Conclusions:**

The haplotype association analysis corroborated the results of the linkage study that the telomeric end of BTA18 harbors a gene responsible for BCSE and further refines the BCSE region to a 6.82 Mb interval ranging from 56.05 Mb to 62.87 Mb on BTA18.

## Introduction

Bilateral convergent strabismus with exophthalmos (BCSE) is a heritable eye defect that ccurs in many cattle breeds, e.g., Jersey, German Fleckvieh, German Holstein, and German Brown [1-4]. The incidence of BCSE was estimated to be 0.9% in German Brown cattle [2]. This eye defect is characterized by a progressive, bilateral symmetric anterior-medial rotation of the eyes that is associated with a slight to severe protrusion of the eyeballs. This defect can result in complete blindness. In the development of the bilateral convergent strabismus, a defect in the lateral rectus muscle and the retractor bulbi muscle of the eye or in their appendant nerves (*Nervus abducens* and *Nervus oculomotorius*) might be involved. Histopathological examination of the nuclei of abducens nerves showed significant differences between BCSE-affected and unaffected cows in the number of nerve cells. BCSE-affected animals had a decreased number of nerve cells in both nuclear regions (*Nuclei n. abducentis dexter* and *sinister*), and this may be related with paresis of the *M. rectus lateralis* and the lateral parts of *M. retractor bulbi*, which is also involved in lateral eye movement [5]. The histomorphological examination of the lateral and medial rectus muscles of BSCE-affected cows revealed “ragged red fibers,” which are indicators for defects in the respiratory chain of muscles [6].

The defect sometimes causes changes in the behavior of the affected animals such as aggressiveness, shying, and panic in everyday situations. The first signs of BCSE can appear as early as the age of six months, but most of the affected animals are not noticed before first breeding. This eye anomaly is incurable [1].

In a previously performed whole genome scan using multipoint non-parametric linkage and haplotype analysis in a total of 159 German Brown cattle, we identified a genomic region harboring a locus responsible for BCSE on bovine chromosome 18 (BTA18) [7]. We mapped this BCSE locus to a 6.83 cM interval (MARC-USDA linkage map) on the telomeric end of BTA18 between the microsatellites, *BMS2785* (72.01 cM) and *BM6507* (78.84 cM), using linkage and haplotype analysis. The Zmean and LOD score peaked at marker *DIK5109* (77.60 cM) [5]. This BCSE region corresponds to a 7.77 Mb interval between 55.23 Mb (*BMS2785*) and 63.0 Mb (*BM6507*). These marker positions were determined using BLAST analysis for Btau_4.0 (*Bos taurus* genome assembly 4.0).

We could identify misinnervation syndromes in humans with similarities in pathology and clinical features to BCSE in cattle. Progressive external ophthalmoplegia (PEO), Duane retraction syndrome (DRS), and congenital fibrosis of the extraocular muscles (CFEOM) belong to this group of diseases in humans. PEOs are characterized by slowly progressive bilateral immobility of the eyes accompanied by ptosis. The three candidate genes, *POLG* [8], *ANT1* [9], and *C10orf2* [10], for PEO were ruled out as responsible for BCSE [1,11]. CFEOM [12] and DRS [13,14] belong to a group of congenital cranial nerve dysinnervation disorders (CCDD) affecting the eye, eye lid, and/or facial movement [15]. The various forms of CFEOM [12] result from dysinnervation of the oculomotor nerve innervated ocular muscles and /or trochlear nerve innervated ocular muscles. Genes or loci causing the CFEOM phenotypes include *KIF21A* (*CFEOM1*) on centromeric HSA12q12 [16,17], *ARIX* (*CFEOM2*) on HSA11q.13.3-q13.4 [18], *CFEOM3* on HSA16q24.2-q24.3 [19], and *CFEOM3A* on HSA12p11.2-q12 [20]. The bovine syntenic regions for these genes or loci are on BTA5, 9.7 Mb distally of the Quantitative Trait Locus (QTL) for BCSE (*KIF21A*), on BTA15 at 51.34 Mb (*ARIX*), and on BTA18 from 11.5 to 14.0 Mb (*CFEOM3*). The loci for DRS were mapped to HSA8q13 (*DURS1*) [21-23] and HSA2q31 (*DURS2*) [24,25]. The orthologous bovine loci are on BTA14 between 30.2 and 30.7 Mb (*DURS1*) and BTA2 between 14.7 and 21.3 Mb (*DURS2*). Therefore, none of these loci or genes identified for CCDD in humans are mapping within the QTL for BCSE.

**Figure 1:**
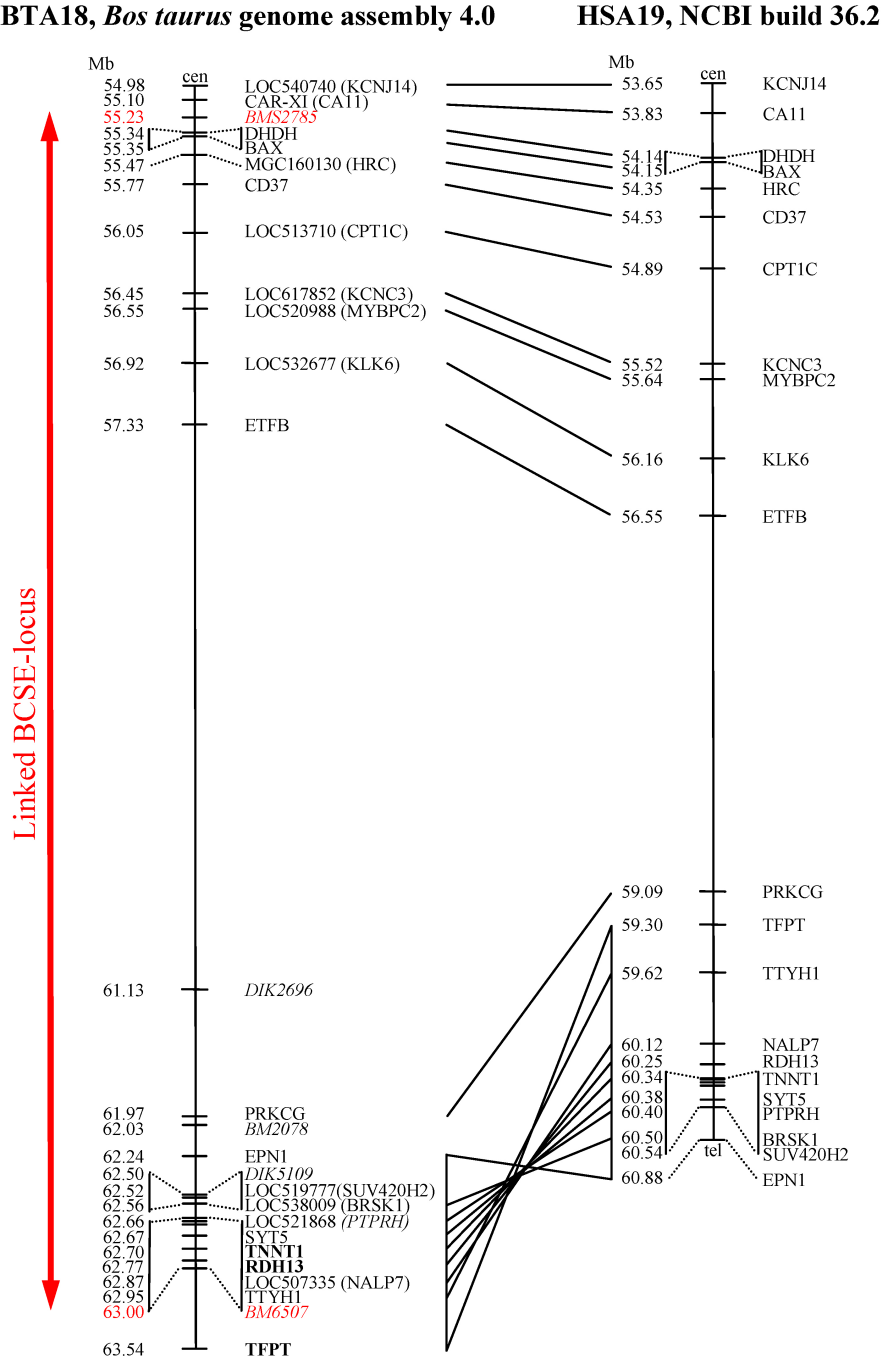
Correspondence between the telomeric region of BTA18 and syntenic region of the human genome on HSA19. The location of the genes and candidate genes (**bold**) in which PCR products were designed are shown. Microsatellites used in the previous linkage study are printed in *italics*. The linked BCSE locus on BTA18 is marked with a red bar.

Comparison of the gene order on the telomeric end of BTA18 (Btau_4.0) with the corresponding region on HSA19 (NCBI Build 36.2) showed two blocks of synteny (Figure 1). The gene order within the first block from *LOC540740* to *PRKCG* is consistent with the human gene order. The second block between *EPN1* (epsin 1) and *TFPT* (TCF3 fusion partner; Btau_4.0) is inverse  compared to the gene order of the human genome assembly 36.2. In our analysis, we considered the interval from *LOC540740* (54.98 Mb) to *TFPT* (63.54 Mb), which included the linked BCSE region and its flanking regions on BTA18.

The aim of this study was to identify single nucleotide polymorphisms (SNPs) associated with BCSE within the previously determined BCSE region and within the coding sequence of possible candidate genes contained in this region. Candidate genes were chosen due to their expression profile and their proximity to the microsatellite, *DIK5109*.

The first candidate gene, troponin T type 1 (*TNNT1*), is located about 200 kb proximal of DIK5109 at 62.50 Mb. The protein product of *TNNT1* is a component of the thin filament of the sarcomere and has the function to prevent actin-myosin interaction in resting muscle. *TNNT1* is highly expressed in skeletal muscles [26]. The second candidate gene, retinol dehydrogenase 13 (*RDH13*), is located in close vicinity to *TNNT1* at 62.70 Mb. *RDH13* belongs to the short-chain dehydrogenases/reductases (SDR) family and is mostly expressed in cranial nerve tissue and in the retina where it was detected in the inner segment of the photoreceptor cells [27]. Mutations causing strabismus have not yet been reported, but related genes such as *RDH5* and *RDH12* were shown to cause *fundus albipunctatus* and retinal dystrophy in human, which can be accompanied by strabismus [28,29]. The third candidate gene, TCF3 fusion partner (*TFPT*), is ubiquitously expressed mainly in the brain, hematopoietic cell lines, and eye tissue.

## Methods

### Animals, phenotypic data, and DNA/RNA extraction

For our analyses, we collected blood samples from 96 unrelated German Brown cows. Of these animals, 48 were affected by BCSE and showed third or fourth stage BCSE where more than 50% of the eye was filled with sclera [30]. The other 48 German Brown cows were unaffected and more than six years old. Thus, these animals are very unlikely to develop the BCSE phenotype. Genomic DNA from EDTA blood samples was extracted using the QIAamp 96 Spin Blood Kit (Qiagen, Hilden, Germany).

For cDNA analysis, we took biopsies from the retina, *N. opticus*, and ocular muscles (*M. rectus lateralis* and *M. retractor bulbi*) of three unaffected and three severely affected cows (BCSE stage 3) [30]. These samples were taken 15–30 min after the cows were slaughtered.

Tissue samples were conserved using RNAlater solution (Qiagen). RNA was extracted from the ocular tissues using the Nucleospin RNA II-Kit (Macherey-Nagel, Düren, Germany) and transcribed into cDNA using SuperScript III Reverse Transcriptase (Invitrogen, Karlsruhe, Germany).

### Gene structure, single nucleotide polymorphisms, polymerase chain reaction, and DNA sequencing

#### Bioinformatic cDNA analysis

**Figure 2:**
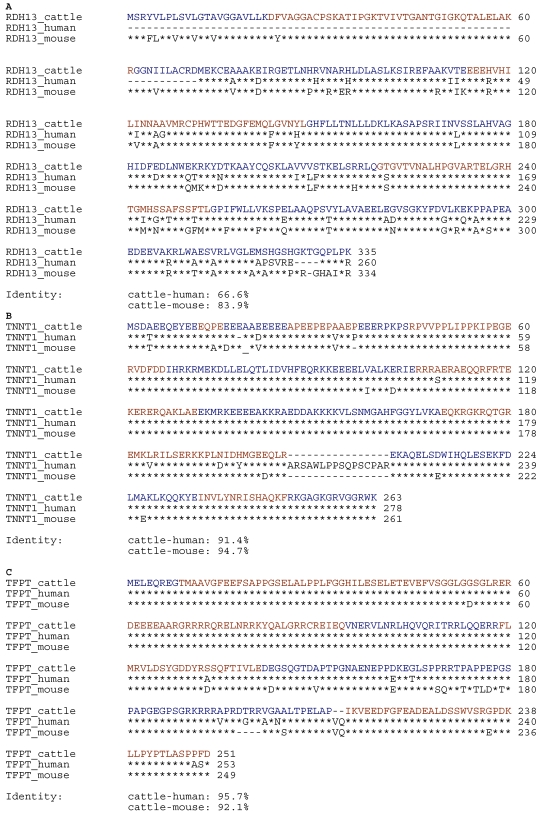
Alignment of RDH13, TNNT1 and TFPT proteins with known human and mouse orthologs. **A**: Shown are the protein sequences of RDH13 for cattle, man and mouse. **B**: Shown are the protein sequences of TNNT1 for cattle, man and mouse. **C**: Shown are the protein sequences of TFPT for cattle, man and mouse. Bovine protein sequences were derived from our analyzed coding sequences, which were similar to the published bovine protein sequences (NP_001068813.1, NP_776899.1, and NP_001068742.1). The sequences were derived from GenBank entries with the accession numbers NP_612421 (human RDH13), NP_780581 (mouse RDH13), NP_003274 (human TNNT1), NP_035748 (mouse TNNT1), NP_037474 (human TFPT), and NP_076013 (mouse TFPT). Identical residues are indicated by asterisks beneath the alignment. The exons are labeled by different colors.

For cDNA analyses of the candidate genes, we searched the cattle expressed sequence tag (EST) archive for ESTs and the bovine genome for annotated genes by cross-species BLAST searches with the corresponding human reference mRNA sequences for *TNNT1* (NM_003283), *RDH13* (NM_138412) and *TFPT* (NM_013342). Table 1 gives an overview of the structure of these human genes and their orthologs in *Bos taurus*. In addition, we verified the sequence homology between the proteins of the three candidate genes in cattle, mouse, and human using the ClustalW alignment program (Figure 2).

**Table 1:** Candidate genes.

**Gene**		***Homo sapiens* (36.2)**				***Bos taurus* (4.0)**				
		**HSA**	**DNA (bp)**	**mRNA (bp)**	**Number of exons**	**BTA**	**DNA (bp)**	**mRNA (bp)**	**CDS (bp)**	**Number of exons**
*TNNT1*	troponin T type 1 (skeletal, slow)	19	16378	980	14	18	9366	887	9 - 800 (exon 1-exon 13)	13
*RDH13*	retinol dehydroge-nase 13 (all-trans/9-cis)	19	25191	2006	8	18	16985	3025	119 - 1126 (exon 1-exon 7)	7
*TFPT*	TCF3 (E2A) fusion partner (in childhood leukemia)	19	8707	1077	7	18	7644	879	62 - 817 (exon 1-exon 6)	6

An overview about the structure of the candidate genes, *TNNT1*, *RDH13*, and *TFPT* of *Homo sapiens* in comparison to *Bos taurus* is given.

We found a bovine EST (EE371552), isolated from muscle tissue with 89% identity to the human *TNNT1* mRNA sequence, and the bovine mRNA of *TNNT1* (NM_174474) with an identity of 90% to human *TNNT1* mRNA (NM_003283).

For *RDH13*, we found two overlapping bovine ESTs (DV925005 and DV828503), which cover 77% of the human mRNA sequence with an identity of 88% and the bovine mRNA of *RDH13* (NM_001075345). The first EST (DV925005) was isolated from the skin of an embryo and the second (DV828503) from fetal pons.

We found three overlapping bovine ESTs (DV851209, CO881320, and CO873631) that were isolated from brain tissue covering the whole human *TFPT* mRNA sequence with an identity of 86%. Furthermore, we identified the bovine *TFPT* employing a genomic BLAST analysis with the bovine mRNA sequence (NM_001075274).

We amplified the cDNA sequence corresponding to the open reading frames (ORF) of the three candidate genes. We used the ESTs and the annotated gene information for primer design with Primer3 software (Table 2).

**Table 2:** cDNA PCR primers.

**Gene**	**Target**	**Primer**	**Primer location**	**Primer sequence (5′-3′)**	**Annealing Temperature (°C)**	**Product Size (bp)**
*TNNT1*	cDNA	TNNT1ex1–9_F	Exon 1	GCCGAAGAGCAAGAATATGA	58 °C	492
		TNNT1ex1–9_R	Exon 9	GACCAGATAACCCCCAAAAT		
		TNNT1ex8–13_F	Exon 8	GCTTCAGAACCGAGAAGGA	58 °C	533
		TNNTex8–13_R	Exon 13	CCCAGATGGACACACACC		
		TNNT_ex4_F_cDNA	Exon 1/2	AGAGGAGCAGCCCTGAAGA	60 °C	172
		TNNT_ex4_R_cDNA	Exon 6	ATGTCATCGAAGTCCACACG		
*RDH13*	cDNA	RDH13_cF1	Exon 1	GCGCTCTAGGTGCAGACTCCG	60 °C	714
		RDH13_cR1	Exon 5	CTCAGCTCCTTGGTGGAGAC		
		RDH13_cF2	Exon 3	GAAGTCCATCCGAGAGTTCG	60 °C	789
		RDH13_cR2	Exon 7	GGCATGACAGCTAGGTTTGG		
*TFPT*	cDNA	TFPT_ex1_F	Exon 1	GAGCCCGATAAACAGACTCG	60 °C	655
		TFPTex1–2_R	Exon 3	GCTGGAGTCTCCGAGTTATTC		
		TFPTex1–2_F	Exon 2	GTGGGCTTCGAGGAGTTC	60 °C	689
		TFPT_ex6_R	Exon 6	TAGGGCAGCAGTTTGTCTGG		

The PCR primers for the amplification of the cDNA of bovine *TNNT1*, *RDH13*, and *TFPT* are shown.

#### Genomic DNA sequence analysis for single nucleotide polymorphism detection

For these analyses we employed four BCSE-affected German Brown cows and four controls of the same breed. First, we designed exon flanking intronic primer pairs for the genomic amplification of all exons of *RDH13* and the exons of *TNNT1* and *TFPT*, which harbored SNPs detected by cDNA analyses (Table 3). Furthermore, we designed primer pairs for four polymerase chain reaction (PCR) products of these candidate genes for SNP detection within intronic regions (Table 4). To cover the whole region of 8.56 Mb extending between LOC540740 and *TFPT*, we screened 17 more genes for DNA polymorphisms. A total of 21 amplicons was sequenced (Table 4). We used the DNA of eight German Brown cows (four affected and four controls) for SNP development in the three positional candidate genes and the 17 genes evenly distributed over the QTL region.

**Table 3:** PCR-Primers for SNP-genotyping.

**Gene**	**Target**	**SNP**	**Primer**	**Primer sequence (5′→3′)**	**Annealing temperature (°C)**	**Product size (bp)**
*TNNT1*	intron 10	AM930546 ; g.273A>G	TNNT1_SNPex11_F	CAGAGTTGGGGATGGATATG	58	597
			TNNT1_SNPex11_R2	AGACCAGAGGGATGTGTTGG		
	exon 11	AM930555 ; c.359A>G	TNNT1_SNPex11_F	CAGAGTTGGGGATGGATATG	58	597
			TNNT1_SNPex11_R2	AGACCAGAGGGATGTGTTGG		
	exon 13	AM930555 ; c.425A>C	TNNT_SNP_ex13_F2	CTGACACCCCTCCTTCTCCT	58	157
			TNNTex8–13_R	CCCAGATGGACACACACC		
*RDH13*	5′UTR	AM930553 ; c.57A>C	RDH13_ex1_F_gen	GCGCTCTAGGTGCAGACTC	60	381
			RDH13_ex1_R_gen	CCGGAAGCAACTAGACCAAA		
	exon 1	AM930553 ; c.103C>G	RDH13_ex1_F_gen	GCGCTCTAGGTGCAGACTC	60	381
			RDH13_ex1_R_gen	CCGGAAGCAACTAGACCAAA		
	intron1	AM930548 ; g.294C>T	RDH13_ex1_F_gen	GCGCTCTAGGTGCAGACTC	60	381
			RDH13_ex1_R_gen	CCGGAAGCAACTAGACCAAA		
	exon 2	AM930553 ; c.151C>T	RDH13_ex2_F_gen	CCTTGGGTTGTGGGATATTG	60	338
			RDH13_ex2_R_gen	CCAACCACCACCAGGTCTTA		
	intron 2	AM930549 ; g.8G>A	RDH13_ex3_F_gen	TTTTCTCGTCTGGCTCTTCC	60	307
			RDH13_ex3_R_gen	TCTTCACCCAAAGACGGAAC		
	intron 3	AM930550 ; g.137T>C	RDH13_ex4_F_gen	CCAGGTAGTTAACGCCAAGC	58	372
			RDH13_ex4_R_gen	TGCCTTTCTCTGGCTCACTT		
	exon 5	AM930553 ; c.703C>A	RDH13_SNPex5_F	GCCACTTCCTTTTGACGAAC	60	355
			RDH13_SNPex5_R	GTCGGGCCTAAGTGTGTCAT		
	exon 7	AM930554 ; c.491G>C	RDH13_SNPex7_F	GGTTCCTGCATCTGGAATTG	60	381
			RDH13_SNPex7_R	ACTTTCAGCCCAAAGCCTCT		
*TFPT*	exon 2	AM930551 ; c.337A>T	TFPT_SNPex3_F	GGCTTGAGCTGTCCAGTGA	60	387
			TFPT_SNPex3_R	TGCGATTTAGTTCCCTCTGG		
	exon 2	AM930551 ; c.379G>T	TFPT_SNPex3_F	GGCTTGAGCTGTCCAGTGA	60	387
			TFPT_SNPex3_R	TGCGATTTAGTTCCCTCTGG		
	exon 5	AM930552 ; c.176G>A	TFPT_exon6_SNP_genF	CTCTCCCGTCTGCCAGGAT	62	297
			TFPT_exon6_SNP_genR	CTTTCCCTGCTTCCCCTGT		

The PCR-primers for amplification of the genomic sequences of bovine *TNNT1*, *RDH13*, and *TFPT* genes which contain the SNP markers are shown.

**Table 4:** PCR-primers used for SNP development on genomic DNA.

**Gene**	**Position HSA19 (Mb)**	BLAST **hit BTA18 (Mb)**	**Primer name**	**Primer sequence (5′ -> 3′)**	**Primer location**	**Product size (bp)**
*KCNJ14*	53.65	54.98	KCNJ14_F	CCAGGGTTGGTGTGAGAACT	exon 2	342
			KCNJ14_R	GCTCTTCCTACCTCCCTGGT	exon 2	
*CA11*	53.83	55.1	CA11_F	GAAACTTCGTGCCAGGTG	exon 2	938
			CA11_R	CACCAGGGTTCTTACCTTCTC	intron 3	
*DHDH*	54.13	55.34	DHDH_F	AGCTTCACCTGCAGCATC	exon 5	955
			DHDH_R	TCCTTATGCTCCCCCTTC	exon 6	
*BAX*	54.15	55.35	BAX_F	TCAGGGGTGAGTTTGAGGTC	exon 2	556
			BAX_R	GGTCCACCCAAACCAAAGA	intron 3	
*HRC*	54.35	55.47	HRC_F1	TGGTCTGCGAAACTCTCTG	exon 7	530
			HRC_R1	CAGGGACGAGGAGAAATAGTC	exon 8	
			HRC_F2	TAACCTGGCTCCTCTGGTC	intron 5	652
			HRC_R2	GAGCAGAGAGTTTCGCAGAC	exon 7	
*CPT1C*	54.89	56.05	CPT1C_F1	TGGACTTTTTCTGACCGACT	intron 3	960
			CPT1C_R1	GACTCAATGGGCTCACATCT	intron 6	
			CPT1C_F2	GCCATGGAGGACAAAGAGA	exon 15	652
			CPT1C_R2	CAGGGCGAGGCACTGTG	exon 16	
*KCNC3*	55.52	56.45	KCNC3_F	CTCCCCATCCACCTTCTC	intron 2	337
			KCNC3_R	GCTTCTTGCATCCTGTTCTC	intron 2	
*MYBPC2*	55.64	56.55	MYBPC2_F	AAAGATGCTGCTGCCAAG	exon 3	619
			MYBPC2_R	TTCTGCTCAGGAGATAAGATCC	intron 3	
*KLK6*	56.16	56.92	KLK6_F	ACGTTCTCTCCTCCACCAG	intron 2	549
			KLK6_R	GTGCTTGCCCAGGTACAC	exon 4	
*ETFB*	56.55	57.33	ETFB_F1	AACCCCTTCTGTGAGATCG	exon 2	347
			ETFB_R1	CCAGGTGATTCGCTTCTG	exon 3	
			ETFB_F2	CTGGCAGAGAAGGAGAAGG	exon 3	818
			ETFB_R2	CTGTCATCTGCCCTGTTTG	exon 4	
*PRKCG*	59.09	61.97	PRKCG_F	TCTCCGATCCCTATGTGAAG	exon 6	808
			PRKCG_R	AAGTCGTTTCGGGAGGTC	exon 7	
*TFPT*	59.3	63.54	TFPT_F	AGTGGAACAGGAGAGGCAGT	intron 2	448
			TFPT_R	AGTGCTCAGACAAGGGTGTG	intron 3	
*TTYH1*	59.62	62.93	TTYH1_F	AGGAGGAGACTGGCCTTG	exon 7	503
			TTYH1_R	CCTGGTTGCAGAGGAAGTAG	exon 8	
*KIR3DL1*	60.02	62.79	KIR3DL1_F	TGTGTTCTCGAGCCTCTTG	exon 3	1181
			KIR3DL1_R	ATCTGTAGGTGCCCACACTC	exon 4	
*NALP7*	60.14	62.87	NALP7_F	ACTCTGAGACACCCGAAGTG	exon 4	863
			NALP7_R	AGCTGACATTCAGGGTAACG	exon 5	
*RDH13*	60.26	62.77	RDH13_F	AGCCCTCCAGGTGATGTTTA	intron 5	714
			RDH13_R	GGTGTCTGTGAGGGTGTGTG	intron 6	
*TNNT1*	60.34	62.7	TNNT_F1	CGCCGAAGAGCAAGAATA	exon 1	602
			TNNT_R1	AGAGGAGAGAGGGGAGAGG	intron 3	
			TNNT_F2	AAAAGGGCCCCAAATTATTA	intron 10	511
			TNNT_R2	TCCAGAGAAAGAGGGATGAA	intron 12	
*SYT5*	60.38	62.67	SYT5_F	TCACTGGACTATGATTTCCAGAC	exon 4	681
			SYT5_R	AGCGGTCGAAGTCGTACA	exon 6	
*BRSK1*	60.5	62.56	BRSK1_F	CTCAACTCCATCCGCAAC	exon 15	556
			BRSK1_R	GACATCTCCTCGGCAGTG	exon 16	
*SUV420H2*	60.55	62.52	SUV420H2_F1	AAAGTGGCTTCACCATCTTG	exon 4	931
			SUV420H2_R1	TCTCAGCAGCTCCTCGTC	exon 5	
			SUV420H2_F2	GGATGGCCCACTACTTCC	exon 3	493
			SUV420H2_R2	CATGGAATAACGGGTACAGG	exon 4	

The gene, its position on HSA19, BLAST hits on BTA18 (Btau_4.0), F and R primers, primer location, and product size are presented. The annealing temperature for all PCR reactions was 58 °C.

#### Polymerase chain reaction and DNA sequencing

We used 48 BCSE-affected German Brown cows and 48 unaffected cows of the same breed. PCR reactions were performed in a total volume of 30 µl using 2 µl (~20 ng/µl) genomic DNA, 3 µl 10X PCR buffer, 6 µl 10X PCR Enhancer (PeqLab, Erlangen, Germany), 0.6 µl (10 µM) of each primer, 0.6 µl dNTPs (10 mM each), and 0.2 µl (5 U/µl) Taq polymerase (Roche, Mannheim, Germany). The reactions were performed in TProfessional thermocyclers (Biometra, Goettingen, Germany) and started with 5 min initial denaturation at 95 °C followed by 36 cycles at 95 °C for 30 s, optimum annealing temperature (Ta) around 58–60 °C for 1 min, and extension at 72 °C for 45 s. The PCR was completed with a final cooling at 4 °C for 10 min. After purification of the PCR products with MinElute 96 UF Plate (Qiagen), the amplicons were directly sequenced with the DYEnamic ET Terminator Cycle Sequencing kit (GE Healthcare, Freiburg, Germany) on a MegaBACE 1000 capillary sequencer (GE Healthcare). Sequence data was analyzed using the Sequencher 4.7 program (GeneCodes, Ann Arbor, MI).

We analyzed a total of 41 PCR products within 20 genes (Table 2, Table 3, and Table 4). We genotyped all 20 SNPs detected in the cDNA and genomic sequences of the three candidate genes as well as the nine SNPs detected within the additional genes in the BCSE region to obtain a complete sampling from 48 BCSE-affected German Brown cows and 48 unaffected cows of the same breed (Table 5).

**Table 5:** SNP analysis.

**Gene**	**SNP**	**Location**	**Number of**		**Minor allele frequencies (%)**		**HET (%)**	**PIC (%)**	**p (HWE)**
			**cases**	**controls**	**cases**	**controls**			
*KCNJ14*	AM922316 ; g.141C>G	exon 2	32	34	50	39.71	34.9	37.2	0.0165
*DHDH*	AM930537 ; g.351A>C	intron 5	38	34	16.88	30.88	36.1	29.6	0.9928
*BAX*	AM930538 ; g.493T>C	intron 3	43	40	18.6	11.25	25.3	22.3	0.9197
*CPT1C*	AM930539 ; g.569A>G	intron 15	43	43	22.09	36.05	39.5	32.7	0.7016
*PRKCG*	AM930540 ; g.161C>T	intron 6	43	40	32.56	32.5	36.1	34.3	0.1077
*SUV420H2*	AM930545 ; g.129C>T	intron 4	34	41	20.59	20.73	33.3	27.4	0.8861
*SYT5*	AM930544 ; g.71G>A	intron 4	39	36	7.69	16.67	24	18.9	0.2376
*TNNT1*	AM930546 ; g.273A>G	intron 10	43	39	6.98	5.13	9.8	10.8	0.18
*TNNT1*	AM930555 ; c.359A>G	exon 11	33	31	22.73	4.84	21.6	21.3	<0.0001
*TNNT1*	AM930555 ; c.425A>C	exon 13	40	40	23.75	26.25	32.5	30.5	0.233
*RDH13*	AM930553 ; c.57A>C	5′UTR	48	47	39.58	35.11	49.5	35.9	0.579
*RDH13*	AM930553 ; c.103C>G	exon 1	48	46	22.92	29.35	35.1	31.1	0.3876
*RDH13*	AM930548 ; g.294C>T	intron1	32	30	13.75	11.29	41.7	35.9	0.8591
*RDH13*	AM930553 ; c.151C>T	exon 2	46	46	19.57	13.04	30.4	23.6	0.2694
*RDH13*	AM930549 ; g.8G>A	intron 2	46	45	11.96	12.22	22	19	0.745
*RDH13*	AM930550 ; g.137T>C	intron 3	45	40	12.22	21.25	25.9	23.7	0.5842
*RDH13*	AM930553 ; c.703C>A	exon 5	48	45	11.46	21.11	23.7	23.4	0.2257
*RDH13*	AM930547 ; g.113G>C	intron 5	40	38	17.5	23.68	30.8	27.3	0.6181
*RDH13*	AM930547 ; g.194C>T	intron 5	44	43	11.36	20.93	27.6	23.4	0.8409
*RDH13*	AM930547 ; g.333C>T	intron 6	41	43	17.07	19.77	8.7	8	0.9192
*RDH13*	AM930547 ; g.378A>G	intron 6	39	42	14.29	20.24	19.8	25.6	0.6617
*RDH13*	AM930554 ; c.491G>C	exon 7	48	43	7.29	21.28	44	29.9	0.9942
*NALP7*	AM930543 ; g.103T>G	intron 4	29	35	45.35	26.32	23.4	18.6	0.2883
*TTYH1*	AM930542 ; g.365G>A	intron7	29	35	17.24	7.14	23.4	18.6	0.2883
*TFPT*	AM930551 ; c.337A>T	exon 2	7	7	7.14	0	7.1	6.7	0.8898
*TFPT*	AM930551 ; c.379G>T	exon 2	7	7	7.14	0	7.1	6.7	0.8898
*TFPT*	AM930541 ; g.120T>C	intron 2	48	47	8.33	14.89	23.2	18.4	0.2018
*TFPT*	AM930541 ; g.342G>A	intron 3	39	42	2.56	3.57	6.2	5.8	0.7744
*TFPT*	AM930552 ; c.176G>A	exon 5	7	7	7.14	0	7.1	6.7	0.8898

The intragenic single nucleotide polymorphisms (SNPs, n=29) are shown with their number of genotyped cases and controls, minor allele frequencies of genotyped cases and controls, polymorphism information content (PIC), heterozygosity (HET), and test results for Hardy–Weinberg equilibrium (HWE).

### Statistical analyses

A case-control analysis based on χ^2^-tests for genotypes, alleles, and trend of the alleles was performed using the CASECONTROL procedure of SAS/Genetics (SAS, version 9.1.3; Statistical Analysis System, Cary, NC). The ALLELE procedure of SAS was used for estimation of allele frequencies and tests for Hardy–Weinberg equilibrium (HWE) of genotype frequencies. Statistical calculation of pairwise linkage disequilibrium (LD) was performed and pictured using HAPLOVIEW 4.0 [31]. We used the Tagger algorithm r^2^≥0.8 [32] to detect SNPs with strong LD among alleles. Subsequently, the association of haplotypes with BCSE was tested using the HAPLOTYPE procedure of SAS/Genetics.

## Results

### Hardy–Weinberg equilibrium and minor allele frequencies

In total, we developed 29 SNPs within 12 genes. Of these 29 SNPs, 20 were located within the three candidate genes, *TNNT1*, *RDH13*, and *TFPT*. The other nine SNPs were discovered in nine different genes located in the 8.56 Mb interval between LOC540740 (similar to inward rectifier potassium channel*)* and *TFPT*. The genotypic distributions of the 27 genotyped SNPs were in Hardy–Weinberg equilibrium. The SNPs within LOC540740 and exon 11 of *TNNT1* were not in Hardy–Weinberg equilibrium. Thus, these SNPs were not considered in the subsequent association analyses. The results of the tests for HWE, the observed heterozygosity (HET), polymorphism information content (PIC), and minor allele frequencies for the developed SNPs are shown in Table 5.

### Mutation analysis within bovine *TNNT1*, *RDH13*, and *TFPT*

**Figure 3:**
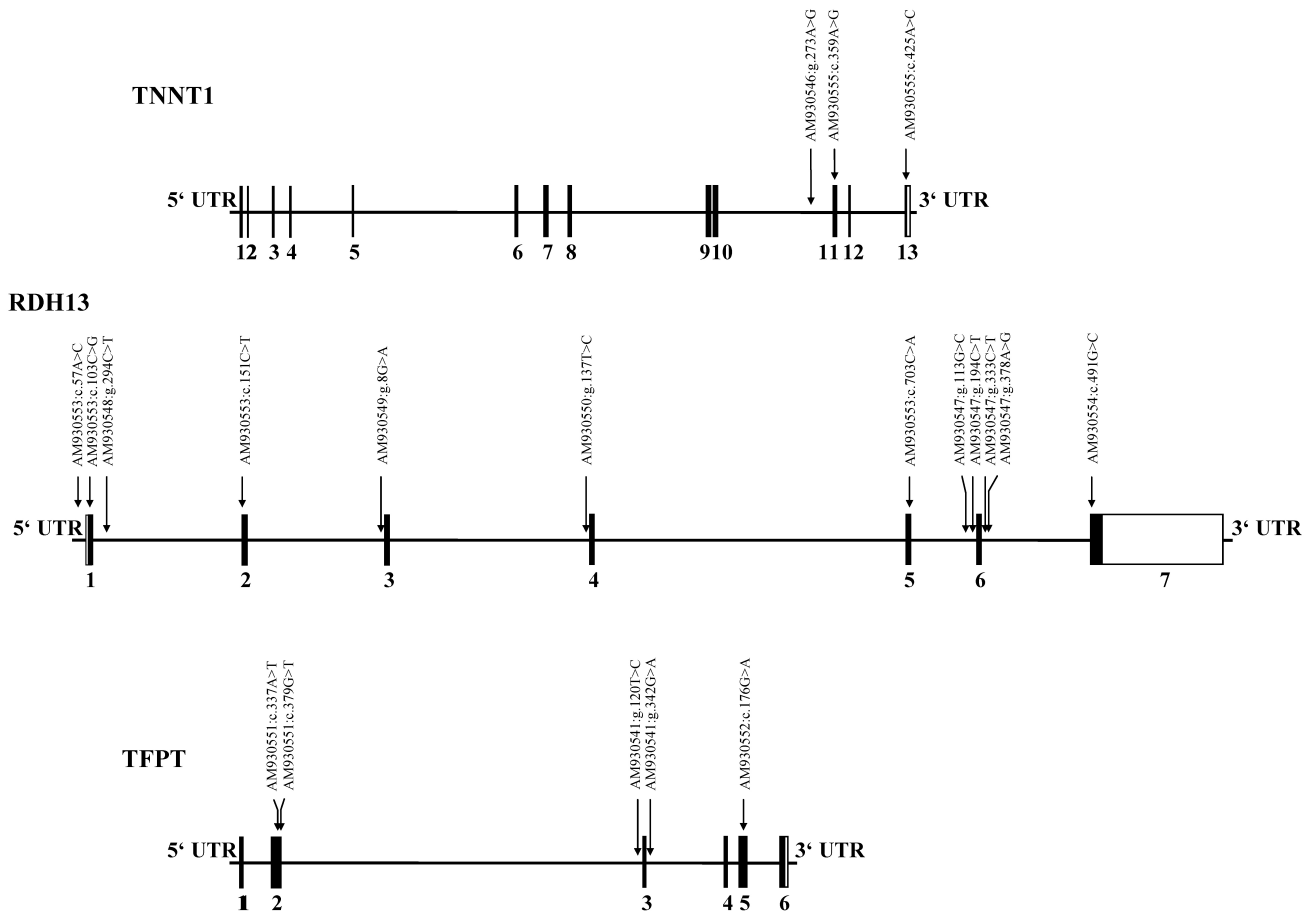
Gene structure and detected single nucleotide polymorphisms within the candidate genes. The gene structure and the detected SNPs within *TNNT1* (9,366 bp), *RDH13* (16,985 bp), and *TFPT* (7,644 bp) are shown. The positions of the SNPs are indicated by arrows. Translated exons are shown as solid boxes and numbered with Arabic numerals. Untranslated regions of exons are shown as open boxes.

**Figure 4:**
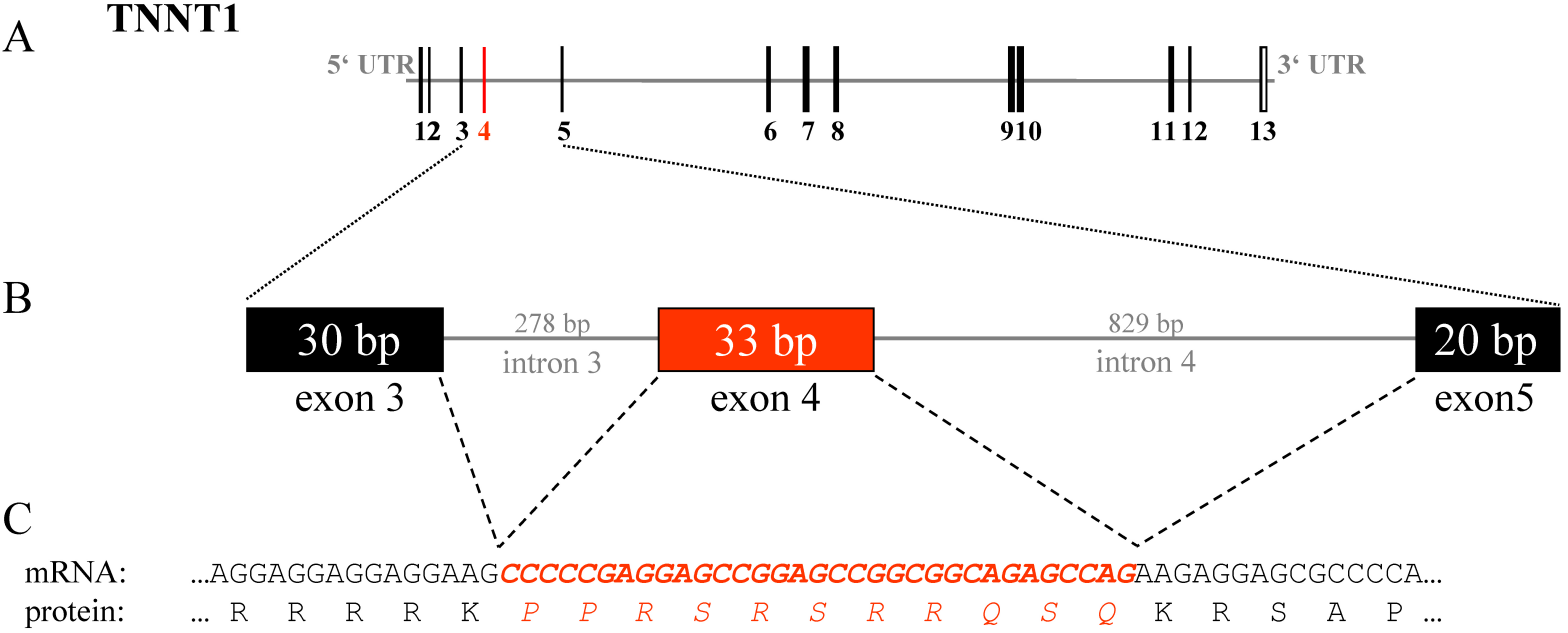
Splice variant of *TNNT1.* **A**: The genomic structure of *TNNT1* is shown. **B**: Detailed illustration of the genomic region from exon 3 to exon 5 is given. **C**: The mRNA sequence of exon 4, their flanking sequences, and the corresponding translated amino acids are pictured. Translated exons are shown as solid boxes. Untranslated regions are shown as gray bars. The exons are numbered by Arabic numerals. The length in base pairs is given for each sequence. Exon 4 is marked in red. The corresponding mRNA sequence and the translation product of exon 4 are printed in *italics*.

We revealed a total of 10 exonic SNPs within the three candidate genes and a new splice variant of *TNNT1* (Table 5). Furthermore, we detected 10 SNPs in the intronic sequences of these candidate genes (Figure 3 and Figure 4).

### TNNT1

Two exonic SNPs are located in the coding sequence of *TNNT1*. One SNP was found in exon 11 and the second in exon 13. Both SNPs did not affect the amino acid sequence. We also identified a deletion of 33 base pairs in the cDNA sequence from eye muscle tissue of all six cows. These 33 base pairs conform to exon 4 of the published bovine mRNA (NM_174474; Figure 4). In contrast, the cDNA isolated from the retina showed that all tested animals were heterozygous for this splice variant. In nerve tissue, all three genotypes were found. In addition, we found one SNP within intron 10.

### RDH13

In *RDH13*, five exonic SNPs were detected. An A>C transversion (AM930555; c.425A>C) is located in the 5′UTR 14 bases upstream of the start codon. A C>G SNP (AM930553; c.103C>G) is located at position 151 of bovine mRNA (NM_001075345) in exon 1. This SNP changes a CGG triplet to a GGG triplet and thus causes an amino acid exchange from arginine to glycine (p.Arg11Gly). This means there is a change from a charged alkaline amino acid to a nonpolar amino acid. The second SNP (AM930554; c.491G>C), which results in an amino acid exchange from glutamine to glutamate (p.Gln233Glu), was found at position 33 of bovine exon 7. This G>C transversion changes a GAG triplet to a CAG triplet, which has the effect that a polar and uncharged amino acid is replaced by an acidic, nonpolar, and charged amino acid in the primary structure of the protein. In addition, we detected two synonymous SNPs in the coding sequence of exon 2 and 5. Within the introns of *RDH13*, we detected one SNP in introns 1, 2, and 3 and two SNPs each in intron 5 and 6 (Table 5).

### TFPT

In the coding sequence of *TFPT*, we identified three exonic SNPs. Two of them are located at positions 2 and 44 of exon 2. Both mutations affect the protein structure. The first exon 2 SNP (AM930551; c.337A>T) is an A>T transversion, which causes an amino acid exchange from threonine to serine (p.Thr9Ser). However, both amino acids are polar, uncharged, and differ in only one ─CH_3_ side chain. The second SNP (AM930551; c.379G>T) in exon 2 alters the protein structure due to a G>T transversion, which changes a GGC triplet to a TGC triplet (p.Gly23Cys). This means that the nonpolar amino acid, glycine, is exchanged with the polar, sulfur-containing amino acid, cysteine. The third exonic SNP found in the ORF of *TFPT* is a synonymous mutation. This G>A SNP (AM930552; c.176G>A) at position 72 in exon 5 changes a CTG to a CTA triplet, which has no effect on the amino acid sequence of *TFPT*. In addition, we identified one SNP in introns 2 and 3 of *TFPT*.

### Association analysis

We detected four SNPs significantly associated with BCSE. These were located in *DHDH*, *CPT1C*, *TNNT1*, and *NALP7* (Table 5 and Table 6). An exonic A>G transition (AM930555; c.359A>G) within *TNNT1* reached significant results in allele and trend test statistics (Table 6).

**Table 6:** Single marker association.

**Gene**	**SNP**	**χ^2^ allele**	**p allele**	**χ^2^ genotype**	**p genotype**	**χ^2^ trend**	**p trend**
*KCNJ14*	AM922316 ; g.141C>G	1.41	0.235	1.58	0.454	1.09	0.296
*DHDH*	AM930537 ; g.351A>C	3.78	0.052	3.79	0.151	3.78	0.052
*BAX*	AM930538 ; g.493T>C	1.75	0.186	2.39	0.303	1.73	0.188
*CPT1C*	AM930539 ; g.569A>G	4.06	0.044	7.51	0.023	3.9	0.048
*PRKCG*	AM930540 ; g.161C>T	<0.001	0.994	0.58	0.749	<0.001	0.994
*SUV420H2*	AM930545 ; g.129C>T	<0.001	0.983	3.78	0.151	<0.001	0.983
*SYT5*	AM930544 ; g.71G>A	2.86	0.091	3.31	0.069	3.31	0.069
*TNNT1*	AM930546 ; g.273A>G	0.24	0.621	2.82	0.244	0.21	0.645
*TNNT1*	AM930555 ; c.359A>G	8.47	0.004	4.74	0.094	4.53	0.033
*TNNT1*	AM930555 ; c.425A>C	0.13	0.715	0.95	0.622	0.11	0.732
*RDH13*	AM930553 ; c.57A>C	0.41	0.524	1.51	0.469	0.43	0.511
*RDH13*	AM930553 ; c.103C>G	1	0.315	2.4	0.301	0.93	0.336
*RDH13*	AM930548 ; g.294C>T	0.19	0.662	2.44	0.295	0.19	0.659
*RDH13*	AM930553 ; c.151C>T	1.43	0.231	1.97	0.374	0.9	0.203
*RDH13*	AM930549 ; g.8G>A	0.003	0.956	1.18	0.552	0.003	0.955
*RDH13*	AM930550 ; g.137T>C	2.51	0.113	2.44	0.295	2.37	0.124
*RDH13*	AM930553 ; c.703C>A	3.2	0.074	2.84	0.241	2.84	0.092
*RDH13*	AM930547 ; g.113G>C	0.91	0.339	1.34	0.513	0.87	0.352
*RDH13*	AM930547 ; g.194C>T	2.94	0.086	3.45	0.177	3.01	0.083
*RDH13*	AM930547 ; g.333C>T	0.2	0.653	0.33	0.85	0.2	0.655
*RDH13*	AM930547 ; g.378A>G	0.002	0.965	1.56	0.459	0.002	0.966
*RDH13*	AM930554 ; c.491G>C	1.61	0.205	1.61	0.445	1.61	0.205
*NALP7*	AM930543 ; g.103T>G	6.31	0.012	5.57	0.062	5.38	0.02
*TTYH1*	AM930542 ; g.365G>A	3.13	0.077	3.61	0.058	3.61	0.058
*TFPT*	AM930551 ; c.337A>T	1.04	0.309	1.08	0.299	1.08	0.299
*TFPT*	AM930551 ; c.379G>T	1.04	0.309	1.08	0.299	1.08	0.299
*TFPT*	AM930541 ; g.120T>C	2	0.158	2.3	0.13	2.3	0.13
*TFPT*	AM930541 ; g.342G>A	0.14	0.711	0.14	0.707	0.14	0.707
*TFPT*	AM930552 ; c.176G>A	1.04	0.309	1.08	0.299	1.08	0.299

The results of association analysis of 29 intragenic SNPs with bilateral convergent strabismus with exophthalmos in German Brown cattle, their χ^2^-test statistics of the case-control analysis, and their error probabilities (p) are presented.

The SNPs within the exons and the exon flanking intronic sequences of *RDH13* showed no significant results from the χ^2^ tests for distribution of genotypes between cases and controls. The χ^2^ test statistics for allelic distributions between cases and controls ranged from 0.003 to 3.20 and their error probabilities from 0.07 to 0.97 for the *RDH13* SNPs. Four exon SNPs clearly failed the threshold of significance. Only the C>A SNP (AM930553; c.703C>A) in exon 5 with an allelic χ^2^ value of 3.20 was close to the threshold of 0.05 (Table 6).

The exonic SNPs of *TFPT* were not genotyped for the complete sample due to their low minor allele frequency (Table 5), and the other intronic SNPs were not associated with BCSE (Table 6).

### Linkage disequilibrium and haplotype association

**Figure 5:**
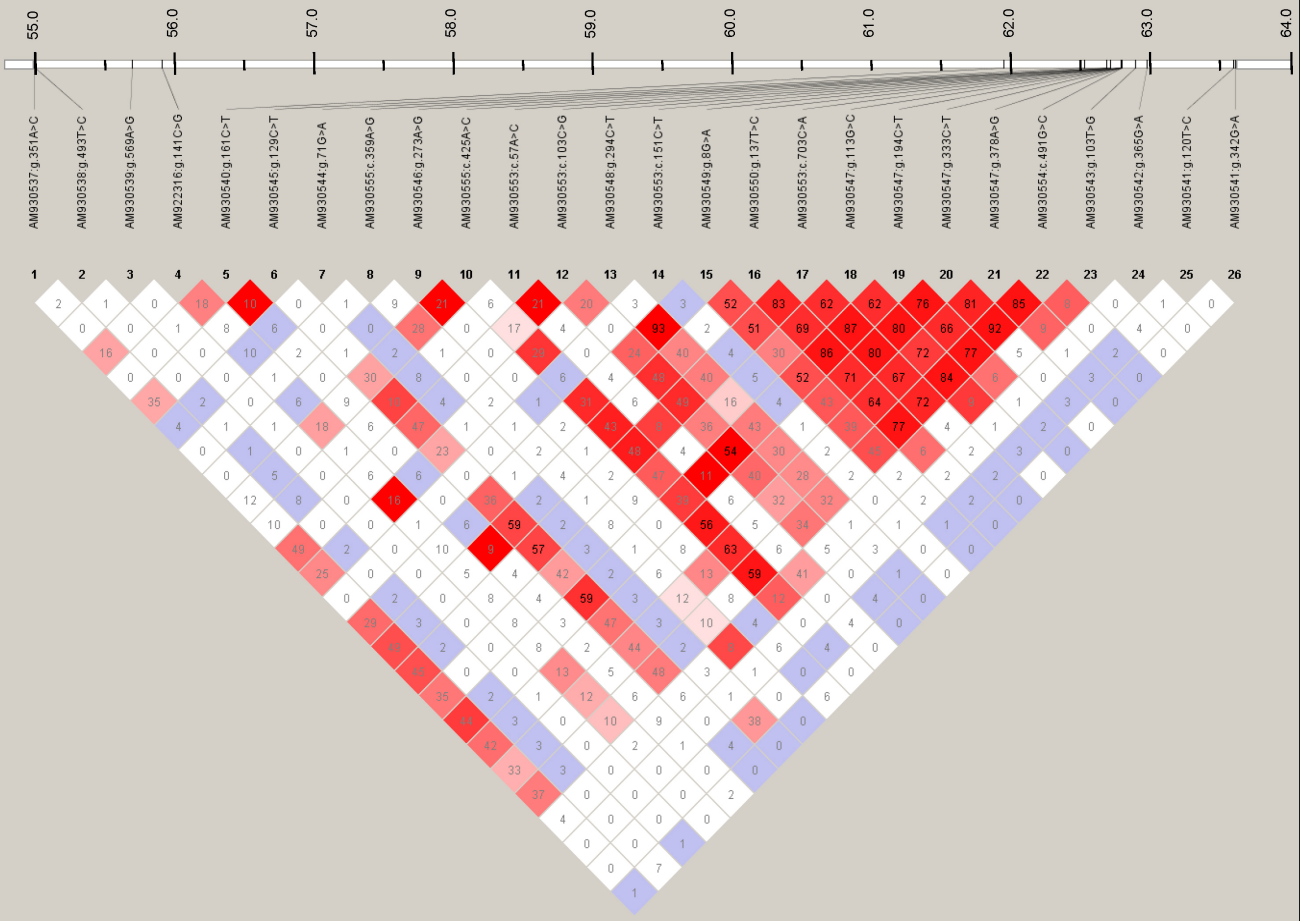
Linkage disequilibria and positions of the single nucleotide polymorphisms on the telomeric end of BTA18. LD coefficients (r^2^) between the SNP pairs are indicated, and the position on BTA18 is pictured. Red fields with black font display r^2^ values greater than 0.50. White and lilac fields display r^2^ values less than 0.15.

The r^2^ values indicated strong linkage disequilibrium (LD) for the SNPs between intron 1 and intron 2 of *RDH13*. By tagging with threshold r^2^≥0.8, we detected five SNPs in *RDH13*, which were representative for the total of 12 *RDH13* SNPs. Therefore, only these five SNPs of *RDH13* were used in the haplotype association analysis. The SNPs within the other genes were not in LD (Figure 5).

We tested the association of haplotypes with BCSE including three to eight SNPs and permutated the number of SNPs that were in Hardy–Weinberg equilibrium. The marker-trait association including five SNPs located in the genes, *CPT1C* (AM930539; g.569A>G), *SYT5* (AM930544; g.71G>A), *RDH13* (AM930553; c.703C>A and AM930547; g.194C>T), and *NALP7* (AM930543; g.103T>G), was significant (χ^2^=54.11, p<0.0001). In total, there were eight different haplotypes of these markers that had a frequency of at least 1% (Table 7). Three individual haplotypes were significantly associated with the affected status and occurred with a frequency of more than 5% in our sample. The A-G-C-C-G haplotype occurred with a frequency of 31.7% in our sample of affected cows and with a frequency of 9.4% in the controls. The A-G-A-T-T haplotype occurred with a frequency of 17.1% in the sample of unaffected cows and with a frequency of 6.1% in the affected cows. The third associated haplotype (G-G-C-C-T) was found with a frequency of 17.0% in our sample of unaffected cows and with a frequency of 7.0% in the affected cows (Table 7). The significantly associated haplotypes spanned the region from *CPT1C* (56.05 Mb) to *NALP7* (62.87 Mb) on the telomeric end of BTA18. Further, two haplotypes adjacent to the proximal and distal region of this aforementioned associated region were tested for association with BCSE. The first haplotype proximally to *CPT1C* consisted of the SNPs (AM922316; g.141C>G, AM930537; g.351A>C, AM930538; g.493T>C), and the second haplotype distally to *NALP7* included the SNPs (AM930542; g.365G>A, AM930541; g.120T>C, AM930541; g.342G>A). Both adjacent haplotypes did not show significant results in marker-trait association tests with BCSE (χ^2^=11.7, p =0.07 corresponds to the first haplotype proximally to the BCSE-associated region and χ^2^=7.3, p=0.12 corresponds to the second haplotype distally to the BCSE-associated region).

**Table 7:** Haplotype association.

**SNP**					**Haplotype**	**Frequency total (%)**	**Standard error**	**Frequency (%)**		**χ^2^**	**p**
**1**	**2**	**3**	**4**	**5**				**controls**	**cases**		
A	G	C	C	T	A-G-C-C-T	28.02	0.0325	21.63	33.94	3.6	0.0575
A	G	C	C	G	A-G-C-C-G	23.2	0.0305	9.39	31.68	14.01	0.0002
G	G	C	C	G	G-G-C-C-G	10.87	0.0187	14.26	11.16	1.12	0.2889
A	G	A	T	T	A-G-A-T-T	10.03	0.0176	17.08	6.17	6.79	0.0092
G	G	C	C	T	G-G-C-C-T	9.79	0.0139	16.95	6.96	6.35	0.0117
A	A	C	C	T	A-A-C-C-T	7.73	0.0112	9.93	5.36	1.41	0.2351
G	G	A	T	T	G-G-A-T-T	3.23	0.0109	2.05	2.34	0.67	0.4132
G	A	C	C	T	G-A-C-C-T	3.09	0.0106	2.55	2.38	0.26	0.6126

Frequencies of the haplotypes with a frequency of at least 1% in the total sample of 96 German Brown cattle and their standard errors, haplotype frequencies of cases and controls, and their association with BCSE on bovine chromosome 18 are shown. In the "SNP" column, SNP 1=AM930539; g.569A>G within *CPT1C*; SNP 2=AM930544; g.71G>A within *SYT5*; SNP 3=AM930553; c.703C>A within exon 5 of *RDH13*; SNP 4=AM930547; g.194C>T within intron 5 of *RDH13*; and SNP 5=AM930543; g.103T>G within *NALP7*.

## Discussion

We developed a total of 29 intragenic SNPs within an 8.56 Mb region on BTA18 extending from *LOC540740* to *TFPT*. In a previously performed whole genome scan, this region was found to be linked with BCSE in German Brown dairy cattle [7]. Within the genes, *CPT1C* and *NALP7*, two SNPs were significantly associated with BCSE in association tests for single markers. Within *DHDH*, *TTYH1*, and *SYT5*, three more SNPs reached values close to the significance threshold of p=0.05.

Most of the 29 SNPs were detected in the sequences of the potential candidate genes, *RDH1*3, *TFPT*, and *TNNT1*. We identified four missense mutations within the coding sequence of these three genes and also detected a new splice variant of *TNNT1*. Since none of the SNPs within the genes (*RDH1*3, *TFPT*, and *TNNT*) were significantly associated with BCSE in association tests for single markers, these genes are unlikely to be causal for this eye defect. However, the detected polymorphisms may be of importance in studies for other bovine diseases especially the SNPs within *RDH13*. These SNPs could be involved in the genetic pathology of retinal dystrophy or related diseases like the defects reported to be associated to other members of the short-chain dehydrogenases/reductases (SDR) family [27,28].

In cattle, genes influencing the development of strabismus are not yet known. Therefore, neuromuscular eye-disorders in humans with already identified causal genes and even developmental and biological characteristics may be used as candidates for BCSE. However, all potential candidate genes characterized as causal for these syndromes (*PEO*, *CFEOM*, and *DRS*) in humans [11-25] could be ruled out for bovine BCSE because we could not find linkage or these candidate genes did not map in the BCSE-linked region on BTA18.

We employed haplotype analysis to further refine the BCSE region on BTA18. To find the most likely associated haplotype, we permutated the number of SNPs for the different haplotypes within this region and were then able to find a significantly associated haplotype. This haplotype included SNPs from the genes *CPT1C*, *SYT5*, *RDH13*, and *NALP7*. Presence of the haplotype A-G-C-C-G composed of these five SNPs indicated a high probability of an animal to be affected by BCSE later in life whereas the haplotypes A-G-A-T-T and G-G-C-C-T were related with low risk to BCSE. Because the surrounding SNPs did not contribute to the significance of the haplotype association, confirmation has been obtained that this linked BCSE region could be delimited using haplotype analysis. Robustness of the haplotype association was furthermore evident when the surrounding haplotypes were extended with one or three adjacent SNPs from the associated haplotype region. In these cases, the extended haplotypes reached higher χ^2^-test statistics and lower error probabilities as more SNPs of the associated region were included. This result was according to our expectation for this region. In conclusion, the haplotype association refined the BCSE region to a 6.82 Mb interval.

To detect the gene responsible for bovine BCSE, further SNPs have to be developed within this BCSE region spanning from 56.05 Mb to 62.87 Mb on BTA18. Haplotype analysis may then be a valuable tool to determine the most likely BCSE causing gene.

Particularly, SNPs within potential candidate genes like *CPT1C* will be considered. *CPT1C* is located at 54.89 Mb on HSA19 and specifically expressed within the endoplasmic reticulum (ER) in neurons of the brain [33]. Expression was also detected in the retinal pigment epithelium [34]. The function of this gene is not yet clearly defined. *CPT1C* is believed to modulate the palmitoyl-CoA pool associated with the ER and therefore to regulate the synthesis of ceramide and sphingolipids. Ceramide and sphingolipids are important for signal transduction, modification of neuronal membranes, and brain plasticity [35-37]. Since one of the BCSE-associated SNPs is located within intron 15 of bovine *CPT1C* at 56.05 Mb on BTA18, this gene may be a candidate for BCSE.
